# Sentinel lymph node biopsy after neoadjuvant chemotherapy for breast cancer: retrospective comparative evaluation of clinically axillary lymph node positive and negative patients, including those with axillary lymph node metastases confirmed by fine needle aspiration

**DOI:** 10.1186/s12885-016-2829-5

**Published:** 2016-10-18

**Authors:** Yue Yu, Ning Cui, Heng-Yu Li, Yan-Mei Wu, Lu Xu, Min Fang, Yuan Sheng

**Affiliations:** 1 Department of Breast and Thyroid Surgery, Changhai Hospital, the Second Military Medical University, 168 Changhai Road, Yangpu District, Shanghai, 200433 China; 2 Department of Breast and Thyroid Surgery, Shangqiu First People’s Hospital, Shangqiu, Hernan China

## Abstract

**Background:**

To evaluate the accuracy of sentinel lymph node biopsy (SLNB) after neoadjuvant chemotherapy (NAC) in breast cancer patients with axillary lymph node (ALN) metastasis.

**Methods:**

A total of 122 patients with operable breast cancer were enrolled in this single-center retrospective study. Eighty patients were clinically diagnosed with a positive axillary lymph node (ALN) via imaging or physical examination (including 66 patients with biopsy-proven metastasis). The other 42 cases had a clinically negative ALN. After four sessions of neoadjuvant chemotherapy, patients were assigned to an ALN-positive or -negative group. The identification rate (IR) and false negative rate (FNR) were determined in the ALN-negative group.

**Results:**

ALN changed from positive to negative after NAC in 48 patients. Among them, 46 had at least one SLN resected (total IR = 95.8 %). Eight of the 46 SLN-negative patients had pathologically confirmed metastasis of at least one non-SLN (FNR = 36 %). Fifty-five of the 56 patients with a biopsy-proven negative ALN remained ALN negative. Furthermore, 54 of the 56 patients had at least one SLN resected (IR =98.2 %). Three SLN-negative patients of the 54 had at least one positive non-SLN (FNR = 10.7 %).

**Conclusions:**

Due to its high FNR, post-NAC SLNB is not recommended for breast cancer patients with ALN metastasis confirmed by biopsy, though their ALN may become negative after NAC. However, for operable breast cancer with negative ALN, post-NAC SLNB is feasible if the ALN remains clinically negative after NAC.

**Trial registration:**

Retrospective evaluation.

## Background

Sentinel lymph node (SLN) biopsy (SLNB), once used for early-stage breast cancer, has gradually become accepted in cases of operable breast cancer after neoadjuvant chemotherapy (NAC). However, for post-NAC breast cancer patients, whether a SLNB can accurately predict axillary lymph node (ALN) status is still controversial. Recently, many studies investigating pre- or post-NAC SLNB for breast cancer patients reported inconsistent results [1, 2]. Generally, a SLNB can accurately predict ALN status before NAC, but not after NAC [3, 4].

In 2013, two studies suggested that an SLNB cannot predict ALN for post-NAC breast cancer due to its low identification rate (IR) and high false negative rate (FNR) [2, 5]. Nevertheless, further stratified analysis showed that for clinically ALN-negative breast cancer, post-NAC SLNB could be used to evaluate the state of the ALN, but not for clinically ALN-positive patients. After NAC, pathological complete response (PCR) of the lymph node occurred in 30–70 % of clinically ALN-positive patients [6, 7]. These patients are suitable candidates for SLNB to avoid ALN dissection (ALND) complications such as upper limb edema. Previous studies have defined “clinically ALN-positive” as lymph node enlargement detected by physical examination or imaging. These two methods are not sufficiently accurate to predict ALN metastasis, whereas fine needle aspiration (FNA) can. As far as we know, few studies have included breast cancer patients with biopsy-proven ALN metastasis. We designed the current study to further investigate whether post-NAC SLNB can accurately predict ALN for biopsy-proven ALN-positive breast cancer.

## Methods

### Patients and groups

**Fig 1:**
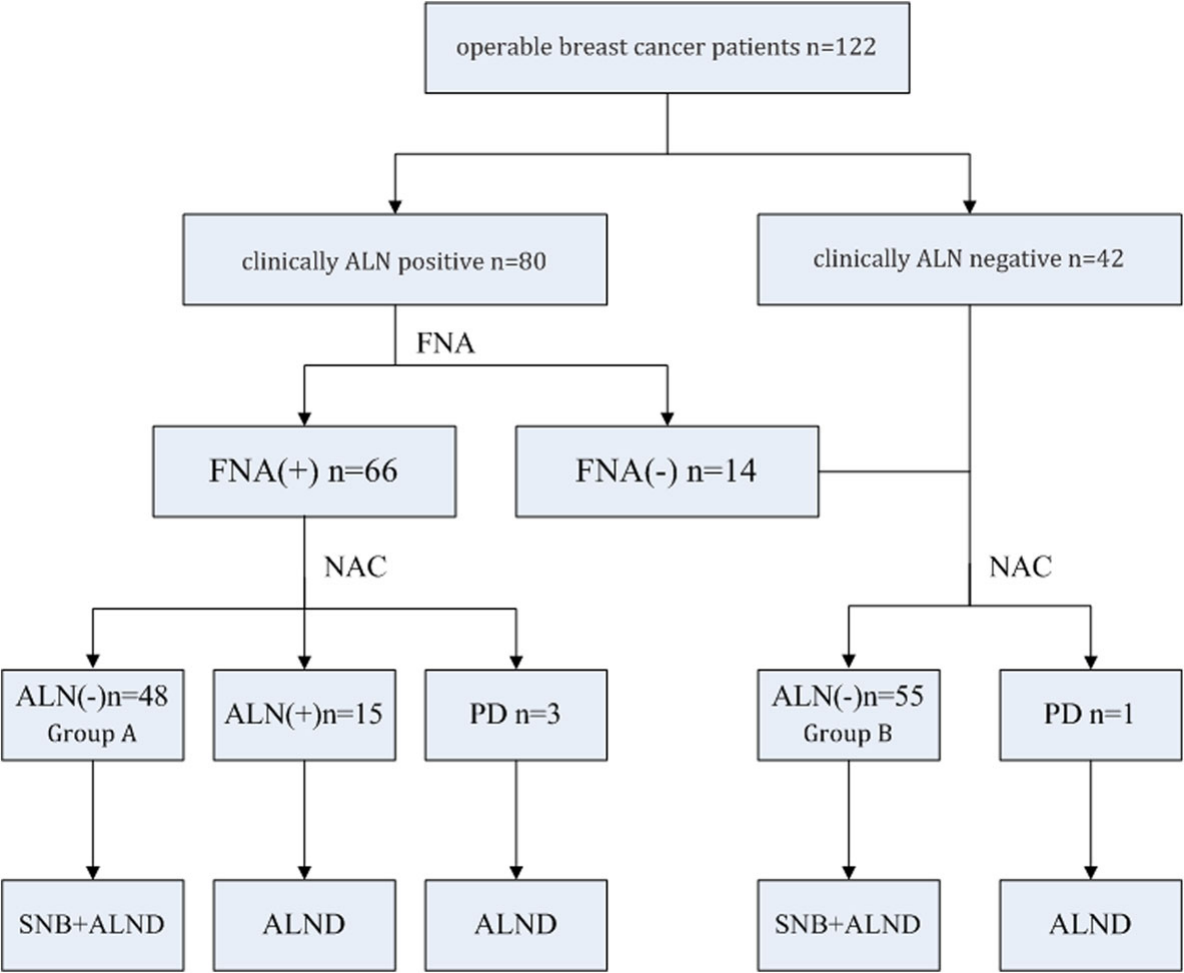
Sentinel node biopsy following neoadjuvant chemotherapy study design. ALN, axillary lymph nodes; FNA, fine-needle aspiration; NAC, neoadjuvant chemotherapy; PD, progression of disease; SNB, sentinel node biopsy; ALND, axillary lymph node dissection

This study was approved by the Ethics Committee of the Second Military Medical University with informed consent from all participants. A total of 122 operable breast cancer patients from the Department of Thyroid and Breast Surgery, First Affiliated Hospital of Second Military Medical University were retrospectively investigated from January 1, 2011 to June 31, 2015. All patients included were females diagnosed with breast cancer based on core needle biopsy with immunohistochemistry (IHC) results. Eighty were clinically ALN-positive breast cancer patients (including 66 biopsy-proven ALN-positive cases). The other 42 were clinically ALN-negative breast cancer patients (Fig. 1). Clinically ALN-positivity refers to lymph node enlargement detected by physical examination or imaging. FNA biopsy includes palpation-guided and ultrasound-guided methods. The exclusion criteria included 1) clinically detected distant metastasis; 2) concomitant malignancies in other organs or a history of previous malignancy; 3) inflammatory breast cancer; 4) uncompleted NAC for any reason; or 5) refusal to participate in this study.

### NAC protocol

All patients enrolled received four sessions of TEC (Doxetaxel 75 mg/m^2^ + Epirubicin 75 mg/m^2^ + CTX 0.6 g/m^2^) NAC. If cancer progress was detected, NAC was ceased and mastectomy and ALND were performed. If ALN remained positive after NAC, mastectomy/breast-conserving surgery and ALND were performed within 2 weeks after NAC. For ALN-negative patients after NAC, we performed mastectomy/breast-conserving surgery with SLNB and ALND. All patients received another two sessions of TEC chemotherapy after surgery. Post-surgery assistant therapy (local radiotherapy + assistant endocrinotherapy/molecular targeted therapy) was provided if necessary.

### Evaluation of NAC efficacy

The tumor and lymph node responses to NAC were evaluated via physical examination and imaging (mainly ultrasound). The response of the primary tumor was assessed using the Solid Tumors System, version 1.1 [8]. Post-NAC ALN-negative breast cancer was defined as the lack of an enlarged lymph node detected on either physical examination or imaging.

### Surgical technique

A radioactive sulfur colloid tracer was not used; therefore, a single tracer technique was employed for all patients. Dye tracing was used for SLNB. SLN was defined as a blue-stained lymph node or lymph node directed by a blue-dyed lymph vessel. Any clinically suspicious or enlarged solid lymph node was also defined as SLN even without blue staining. SLNs were separately submitted for pathological examination after surgery. After removing all SLNs, routine breast surgery and complete level I and II ALN dissection were performed.

### SLN pathology

All SLNs were paraffin embedded for hematoxylin-eosin staining and IHC to assess metastasis. A tumor cell mass larger than 2 mm in diameter was defined as macrometastasis or as positive. IHC was used to evaluate ER, PR and Her-2 expression in primary tumors. When Her-2 showed 2+, FISH was performed for further evaluation.

### Parameters

This study investigated mainly the post-NAC SLN identification rate (IR) and false negative rate (FNR) of breast cancer patients with biopsy-proven ALN metastasis. The following methods were applied to calculate the IR and FNR respectively: IR (%) = cases with successful SLNB/all cases with SLNB × 100 %; FNR (%) = false negative cases/all cases with ALN metastasis × 100 %.

### Statistical analysis

All statistical analyses in this study were performed by the Department of Statistics, Second Military Medical University. The *χ* ^2^ test and Fisher's exact test were used to compare IR and FNR, with *α* < 0.05 indicating statistical significance. The *χ* ^2^ test and fourfold table exact test were used for univariate analysis. The statistics software program used was SAS 9.3.

## Results

### General case information

Sixty-six operable breast cancer patients with initial biopsy-proven ALN metastasis were enrolled in this study. After four sessions of NAC, 48 patients (Group A) became clinically ALN negative and underwent mastectomy/breast-conserving surgery with SLNB and ALND. Eighteen patients remained ALN positive after NAC and underwent mastectomy/breast-conservation surgery and ALND. Additionally, 56 operable breast cancer patients with negative ALN proven by clinical examination and biopsy were also included. One of them presented with ALN progression after NAC and underwent mastectomy and ALND. The other 55 patients (Group B) underwent mastectomy/breast-conservation surgery with SLNB and ALND (Fig. 1).

The average age of the patients in Group A was 50 years. Invasive ductal carcinoma and invasive lobular carcinoma were confirmed in 44 and 4 cases, respectively. Nineteen cases were the luminal A and eight the luminal B molecular subtypes. Ten cases were Her-2 positive. Eleven cases were triple-negative. Twelve (12.5 %) of the 48 patients who completed NAC showed a complete clinical response of the primary tumor. Other general information is shown in Table 1.

**Table 1:** Clinical characteristics of the patients in group A

Clinical characteristics	No. of cases	%
Total	48	
Average age	50.2	
Age at diagnosis		
< 35 yr	18	37.5 %
≥ 35 yr	30	62.5 %
BMI		
≤ 25	38	79.2 %
> 25	10	20.8 %
Clinical tumor volume		
≤ 5 cm	34	70.8 %
> 5 cm	14	29.2 %
Tumor location		
Upper-outer quadrant	30	62.5 %
Lower-outer quadrant	6	12.5 %
Upper-inner quadrant	6	12.5 %
Lower-inner quadrant	4	8.3 %
Nipple area	2	4.2 %
Pathology		
Invasive ductal carcinoma	44	91.7 %
Invasive lobular carcinoma	4	8.3 %
HER2 status		
Positive	16	33.3 %
Negative	32	66.7 %
ER status		
Positive	34	70.8 %
Negative	14	29.2 %
Molecular subtype		
Luminal A	19	39.6 %
Luminal B	8	16.7 %
HER2 positive	10	20.8 %
Triple negtive	11	22.9 %
Tumor response to neoadjuvant chemotherapy		
cPR	26	54.2 %
cCR	12	25 %
cSD	10	20.8 %

In Group A, 46 of the 48 patients had at least one SLN successfully dissected, with a total IR of 95.8 %. For the other two cases, no blue-stained lymph vessel or lymph node was observed or palpated during surgery. A total of 68 SLNs were dissected, with an average of 1.48 SLNs per patient. Of the 46 patients, 32 (66.7 %), 8 (16.7 %), 4 (12.5 %) and 2 (4.2 %) had 1, 2, 3 or 4 dissected SLNs, respectively. A total of 374 lymph nodes were dissected, with an average of 15.6 lymph nodes per patient, as shown in Table 2. Eight cases were SLN positive and non-sentinel lymph node (NSLN) positive. Twenty-four patients were SLN negative and NSLN negative. Eight were SLN negative but NSLN positive. Six were SLN positive but NSLN negative. Fifty-four of the 55 patients in Group B had at least one SLN dissected successfully, with a total IR of 98.2 %. Data on the lymph node status of these 54 cases are shown in Table 3.

**Table 2:** The status of axillary lymph node (ALN) after neoadjuvant chemotherapy in group A

ALN	Non-sentinel node		Total
	Positive	Negative	
Sentinel node			
Positive	8	6	14
Negative	8	24	32
Total	16	30	46

False negative rate 36.4 % (8/(14 + 8);95 % CI, 17–59 %),Overall accuracy 82.6 % ((14 + 24)/46;95 % CI,71–93 %),Sensitivity rate 64 % (14/(14 + 8);95 % CI, 41–83 %)

**Table 3:** The status of axillary lymph node (ALN) after neoadjuvant chemotherapy in group B

ALN	Non-sentinel node		Total
	Positive	Negative	
Sentinel node			
Positive	14	11	25
Negative	3	26	29
Total	17	37	54

False negative rate 10.7 % (3/(25 + 3);95 % CI, 2–28 %),Overall accuracy 94.4 % ((25 + 26)/54;95 % CI, 88–99 %),Sensitivity rate 89.3 % (25/(25 + 3);95 % CI, 72–98 %)

According to the data in Tables 2 and 3, post-NAC SLN-positive patients comprised 30.4 % of all cases with a detected SLN in Group A. Eight of the 46 cases had a negative SLN and at least one metastatic NSLN confirmed pathologically after surgery. As a result, the FNR was 36 % (8/(14 + 8)), with a 95 % CI of 17–59 %. Three of the 54 patients in Group B had a negative SLN and at least one metastatic NSLN confirmed pathologically after surgery, with an FNR of 10.7 % (3/(25 + 3)) and 95 % CI of 2–28 %. No significant correlations were observed between clinical features and the FNR of post-NAC SLNB for breast cancer with ALN metastasis confirmed via biopsy (Table 4).

**Table 4:** False negative rate(FNR) of sentinel node biopsy according to clinicopathological factors

Characteristics	ALN positive No. of cases	SN negative ALN positive No. of cases	FNR	*P*
Total	22	8	36.4 %	
Age				0.402
< 50 yr	8	2	25 %	
≥ 50 yr	14	6	42.9 %	
BMI				0.531
≤ 30	18	6	33.3 %	
> 30	4	2	50 %	
Clinical tumor volume				0.856
≤ 5 cm	16	6	37.5 %	
> 5 cm	6	2	33.3 %	
Tumor location				0.149
Upper-outer quadrant	14	6	42.9 %	
Others	8	2	25 %	
Pathology				-
Invasive ductal carcinoma	22	8	36.4 %	
Invasive lobular carcinoma	0	0		
Vascular invasion				0.402
Yes	8	2	25 %	
No	14	6	42.9 %	
HER-2 status				0.07
Positive	6	4	66.7 %	
Negative	16	4	25 %	
ER status				0.315
Positive	14	4	28.6 %	
Negative	8	4	50 %	
Tumor response to neoadjuvant chemotherapy				0.145
CR	7	2	28.6 %	
PR	13	4	30 %	
ST	2	2	100 %	
No. of SN				0.862
1	8	2	25 %	
2	6	3	50 %	
More than 3	8	3	37.5 %	

## Discussion

Operable breast cancer patients with ALN metastasis confirmed by biopsy may become ALN negative after NAC. For those patients, this study showed that the IR of post-NAC SLNB could reach 95.8 %, which met the recommended IR standard for SLNB by the ASCO guidelines for early-stage breast cancer [9]. However, the FNR of SLNB for patients in Group A was 36 %, much higher than that recommended by ASCO for early-stage breast cancer. For operable breast cancer cases indicated as negative ALN by clinical examination and biopsy, the IR and FNR of SLNB could reach 98.2 % and 10.7 %, respectively, if the ALN remained clinically negative after NAC. These results are consistent with the clinical indications of SLNB.

We performed a literature review of studies using post-NAC SLNB to detect ALN metastasis for clinically positive breast cancer patients. As a result, we found only six studies that included breast cancer patients with ALN metastasis confirmed by biopsy [10–15]. As shown in Table 5, the post-NAC IR ranged from 85.3–96 % and the FNR of SLNB from 8–25 % in these studies. In five previous studies, patients (including both ALN-positive and -negative subjects) underwent SLNB after NAC. The IR and FNR were calculated by summing the cases in the above two groups. In all cases, 50 % of patients still presented as ALN positive after NAC. An expanded study sample without layering could result in discrepancies in the IR and FNR. Kim et al. limited their study population to patients with negative ALN after NAC [15]. Thirty-one of their 120 cases had a negative SLNB result. Nevertheless, Kim et al. did not validate their results by ALND. Kim et al. included only 89 cases (20 SLN-negative cases validated by ALND and 69 SLN-positive cases) to calculate the FNR, which inevitably resulted in a decreased FNR.

**Table 5:** Studies of sentinel node biopsy after neoadjuvant chemotherapy in patients with FNA proved node-positive breast cancer

Authors	Time	No. of cases	Detection rate	FNR
Shen [7]	2007	69	92.8 %	25 %
Newman [8]	2007	54	98 %	8 %
Yagata [9]	2013	95	85.3 %	15.7 %
Park [10]	2013	178	94.9 %	22 %
Boileau [11]	2015	153	87.6 %	8.4 %
Kim [12]	2015	120	96 %	10 %
Present study		48	95.8 %	36 %

The IR of post-NAC SLNB in prior studies ranged from 69–94.9 % [13, 16, 17], lower than that of SLNB for early-stage breast cancer. Some researchers believe that NAC could alleviate ALN lymphadenectasis but also injure the axillary lymph vessels, resulting in lymphatic obstruction. Therefore, tracer and dye usage could not confirm the SLN [18, 19]. The IR of the SLN in our study was higher, close to that for early-stage breast cancer. We attribute this result to our patient selection strategy. All patients included in our study showed an ALN response after NAC. The tumor contained few lymph vessels in these patients, which resulted in limited injury of lymph vessels after NAC. From this point of view, these patients were similar to early-stage breast cancer patients. Thus, it was easier to detect the SLN using a tracer. Meanwhile, we expanded the definition of SLN from a blue-stained lymph node/lymph node directed by a blue-stained lymph vessel to any clinically suspicious lymph node or any enlarged solid lymph node detected during surgery even without blue staining. As a result, we increased the IR of the SLN to 95.8 %, a high value in studies.

The FNR in this study reached the highest value of 36 % compared with other studies. We believed that three factors besides surgical skills could contribute to the elevation of the FNR. First, this result could be associated with the sequence of the ALN response after NAC. Approximately 20 % of patients with a tumor PCR were reported to have a positive ALN, indicating that the tumor CR and lymph CR were not in synchrony [20]. The SLN and NSLN responses to chemotherapy were also not in synchrony. Namely, the SLN and NSLN could have different response sequences after NAC. If the SLN shows PCR but the NSLN does not after NAC, false negative results may result. Under these conditions, the pathology of SLN cannot reflect the reality of the ALN. Second, a high FNR of the SLNB could be related to changes in the lymphatic drainage pathway caused by NAC [18]. The SLN as well as the NSLN may not respond, but lymphatic drainage is altered after NAC. In this setting, SLNB cannot reflect the actual state of the ALN. Third, the high FNR could be associated with patient selection. All patients enrolled were lymph node negative after NAC. Therefore, our FNR was relatively high compared with that found in studies including post-NAC ALN-positive patients [10–14].

In addition, we investigated other factors such as clinical and oncologic features to explain the high FNR of 36.4 % in the present study. No clinicopathologic factors except for Her-2 expression tended to influence the FNR of SLN. Positive results may have been seen if the sample size was increased. We reviewed the literature and found that different molecular subtypes of breast cancer seem to influence the FNR. Yagata et al. reported that Her-2 expression was the major determining factor of the FNR of the SLN among all clinicopathologic factors [12]. Nevertheless, Park et al. stated that triple-negative breast cancer patients had the lowest post-NAC FNR [13]. Molecular subtypes determine the NAC protocol for breast cancer, but whether different protocols lead to a more diverse FNR remains to be confirmed.

Many studies found that the number of lymph nodes identified by SLNB had a strong correlation with the IR and FNR of post-NAC SLNB. One study of SLN FNAC reported an FNR of 18.2 % if one SLN was identified, much higher than that of 4.9 % if two or more SLN were detected [14]. A study by Boughey et al. for lymph-node-positive breast cancer suggested that the FNR of patients with three or more identified SLNs will decrease compared with that of patients with two or fewer identified SLNs (9.1 % *vs* 21.1 %) [4]. An NSABP B-32 study found that for post-NAC breast cancer, the FNR of SLNB decreased with an increasing number of identified lymph nodes. The FNR was 18 % in patients with one identified SLN, 10 % in patients with two identified SLNs, and 7 % in patients with three identified SLNs [21]. Similarly, Hunt et al. reported that for clinical ALN negative breast cancer, the FNR of post-NAC SLNB was higher in patients in whom two or fewer SLNs were identified [22]. As early as 2005, Martin et al. validated that only a single identified SLN could contribute to the elevation of the post-NAC FNR [23]. In our study, the number of identified SLNs was not significantly associated with the FNR, which may have been due to the relatively small sample size in each group. The average number of identified SLNs was relatively small. This may be one reason to account for the increase in FNR.

Patients were classified according to their response to NAC in our study. Post-NAC ALN-positive patients immediately underwent ALND, but the ALN-negative patients underwent SLNB first. This method is more clinically practical and can yield more accurate conclusions. Nevertheless, this was a single-center clinical trial with fewer cases than those in other studies. Multi-center trials with large sample sizes are necessary for a more reliable conclusion.

## Conclusions

In general, in this study, we determined whether post-NAC SLNB was feasible in breast cancer patients with ALN metastasis confirmed by biopsy. Due to the high FNR, breast cancer patients with biopsy-proven ALN metastasis are not recommended to undergo SLNB even if their ALNs became clinically negative after NAC. However, for ALN-negative patients confirmed via clinical examination and biopsy, SLNB is practical if their ALNs remain negative after NAC.

## Abbreviations

ALN: Axillary lymph node
ALND: Axillary lymph node dissection
FNA: Fine needle aspiration
FNR: False negative rate
IR: Identification rate
NAC: Neoadjuvant chemotherapy
NSLN: Non-sentinel lymph node
PCR: Pathological complete response
SLN: Sentinel lymph node
SLNB: Sentinel lymph node biopsy
